# Endocrine regulation of uromodulin in aging

**DOI:** 10.3389/fendo.2026.1905594

**Published:** 2026-09-03

**Authors:** Kerim Mutig, Prim B. Singh, Svetlana Lebedeva

**Affiliations:** 1 Department of Biosciences, School of Medicine, Nazarbayev University, Astana, Kazakhstan; 2 Sirius University of Science and Technology, Sirius Federal Territory, Krasnodarsky krai, Russia; 3 Sechenov First Moscow State Medical University, Moscow, Russia

**Keywords:** aging, kidney stone, renal biomarkers, Tamm-Horsfall glycoprotein, urinary tract infection, uromodulin (UMOD)

## Abstract

Uromodulin (Umod) is the most abundant urinary protein produced by the thick ascending limb (TAL) and distal convoluted tubule (DCT) of the kidney. Its physiological functions range from the protection against urinary tract infections (UTIs) and prevention of urinary stones to the modulation of ion transport along the distal nephron. Apart from the apical release into urine, Umod is delivered to the renal interstitium and serum via basolateral secretion to fulfil immunoregulatory tasks. Mutations and polymorphisms in the *Umod* gene have been associated with kidney and cardiovascular diseases, whereas serum and urinary Umod levels are emerging as biomarkers of renal and cardiovascular risks and conditions. Aging is associated with a gradual decline in both serum and urinary Umod levels, which may contribute to increased risk of UTI and kidney stones and decreased urinary concentration ability in older adults. The present review synthesizes the available information on endocrine regulation of Umod production and release during healthy and pathophysiological aging to specify translational perspectives of targeting the Umod biology and assist by interpretation of serum and urinary Umod levels as biomarkers of renal and cardiovascular health.

## Graphical Abstract

**Figure:**
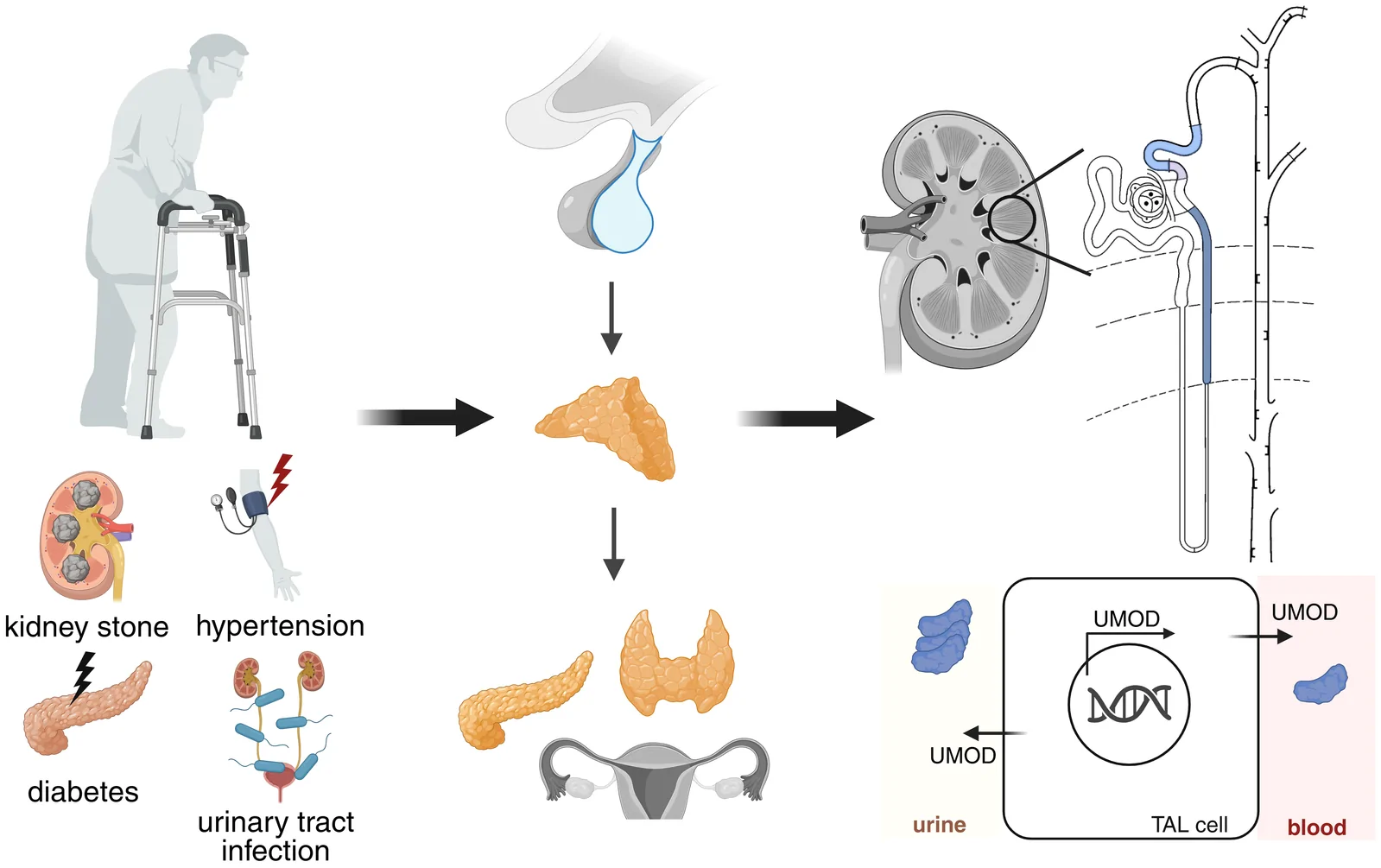

## Introduction

## Uromodulin biology and functions

The history of uromodulin (Umod) discovery started in 1873, when Italian physician Carlo Rovida described a substance produced by renal tubular cells and involved in formation of hyaline casts in the tubular lumen, which he named cilindrina (1, 2). In 1950, Igor Tamm and Frank Horsfall isolated an abundant glycoprotein from human urine and described it as a mucoprotein inhibiting viral hemagglutination (3). For decades this mucoprotein was known as the as Tamm-Horsfall protein (THP). In 1985, A.V. Muchmore and J.M. Decker identified an 85-kilodalton glycoprotein with immunosuppressive activity, highly abundant in urine of pregnant women, and named this protein uromodulin (4). Technical advances in molecular biology and genetics permitted to demonstrate the complete identity of THP and Umod and to identify Umod as the major component of tubular hyaline casts (5, 6). Since then, the term uromodulin has been established as the preferred protein name for better compatibility with its genetic origin (*UMOD* gene) and one of its key physiological functions, namely immunomodulation.

Umod is produced by epithelial cells of the distal nephron of the kidney. The principal sites of Umod synthesis are the medullary and cortical portions of the thick ascending limb (TAL), with minor contribution of the downstream distal convoluted tubule (DCT) (7, 8). Notably, Umod expression is missing in specialized cells of cortical TAL constituting the macula densa (7). Unlike TAL cells with high ion transport activity, MD cells perform critical regulatory tasks for the tubuloglomerular feedback and therefore exhibit a distinct phenotype (9, 10).

With the daily urinary excretion ranging between 30 and 90 mg, Umod is the most abundant urinary protein in healthy individuals (11, 12). The release of Umod into the urine occurs via a proteolytic cleavage of a membrane-anchored precursor by hepsin. The hepsin-mediated Umod cleavage enables assembly of Umod molecules into filamentous homopolymers by releasing the inhibitory external hydrophobic patch (EHP) site and thereby unmasking the zona pellucida domain driving Umod polymerization (13, 14). Apart from the apical release into the urine, Umod is also secreted at the basolateral side and found in the serum. The molecular mechanism of basolateral Umod secretion is distinct from the hepsin-mediated cleavage and produces a larger Umod variant retaining EHP and, for this reason, not capable of polymerization (15).

Umod functions were studied in different physiological contexts including the urinary antimicrobial defense, inhibition of calcium oxalate stone formation, immunomodulation, as well as reabsorption of salt and divalent cations along the distal nephron (16, 17). The antimicrobial defense provided by Umod relies on its high-mannose N-glycosylation site (Asn275) acting as a decoy for the fimbrial adhesin H (FimH) lectin located on the type 1 fimbriae (pili) of uropathogenic *Escherichia coli* (UPEC) to mediate bacterial invasion into the urothelium (18). Umod polymerization occurring immediately after its proteolytic shedding from the cell membrane promotes encapsulation and clearance of UPEC with the urine flow (19). Thus, the polymerizing Umod variant is predominant in the urine and acts as a critical defense factor against UPEC (20).

Apart from the antimicrobial effect, Umod prevents formation of calcium oxalate kidney stones by three complementary mechanisms. Umod contains two calcium-binding motifs and is able to bind free calcium ions thereby limiting Ca^2+^ availability for crystallization. Moreover, binding of Ca^2+^ by these motifs confers a functional Umod conformation enabling coating of microscopic calcium-oxalate crystals and blockade of crystal aggregation (16, 21). Finally, Umod has been shown to facilitate divalent cation reabsorption via transient receptor potential vanilloid 5 (TRPV5) residing in the apical membrane of DCT and connecting tubule (22).

While the apical release of polymerizing Umod variant protects against UTI and urinary stones, the basolateral secretion of non-polymerizing Umod variant serves for local and systemic immunomodulation. Both polymerizing and non-polymerizing Umod suppress activation of neutrophils thereby exerting anti-inflammatory effects in the urinary tract and kidney tissue, respectively (23, 24). Accordingly, Umod-deficient mice displayed increased numbers of neutrophils in the kidney, liver, and circulation (25). Compared to the wild-type phenotype, Umod-deficiency was associated with a stronger kidney damage in a mouse model of acute kidney injury (AKI) (26). Umod-deficiency may lead to exaggerated renal and systemic inflammatory responses due to enhanced renal production of interleukin-17 (IL-17) and IL-23 (25, 26). Apart from inhibitory effects on neutrophils, Umod has been shown to activate mononuclear phagocytes such as macrophages or dendritic cells by triggering the Toll-like receptor 4 signaling (27). Umod can also induce IL-1β secretion by monocytes via activation of the NLR family pyrin domain containing 3 inflammasome (28). These effects are elicited in the urinary tract by the polymerizing Umod, which can also act as a damage-associated molecular pattern (DAMP) to promote the immune response against UPEC (29). Thus, the non-polymerizing Umod variant serves to dampen excessive inflammation in the kidney tissue, whereas the polymerizing Umod variant promotes UPEC destruction and phagocytosis by immune cells. Circulating non-polymerizing Umod exerts systemic antioxidant effects via inhibition of the transient receptor potential cation channel, subfamily M, member 2 (TRPM2) (30). Circulating Umod may also protect against vascular calcification by interfering with pro-inflammatory cytokine signaling (31).

In addition to the extracellular functions, Umod fulfils intracellular tasks associated with the transport activity of TAL and DCT. Experiments in Umod-deficient mice suggested that Umod promotes activating phosphorylation and apical trafficking of the key distal salt transporters, the Na^+^-K^+^-2Cl^-^ cotransporter (NKCC2) of TAL and the Na^+^-Cl^-^ cotransporter of DCT (7, 8, 32). Another study in hepsin-deficient mice with impaired apical Umod cleavage linked Umod accumulation within the luminal membrane to NKCC2 hyperactivity (33). Umod has been also shown to facilitate the luminal potassium recycling in TAL via the renal outer medullary potassium channel (ROMK), which is critical for the proper transport function of NKCC2 (34). Notably, decreased urinary Umod levels were reported in patients with the type 1 or type 2 Bartter syndrome variants caused by loss-of-function mutations in NKCC2 or ROMK (35, 36).

**Figure 1:**
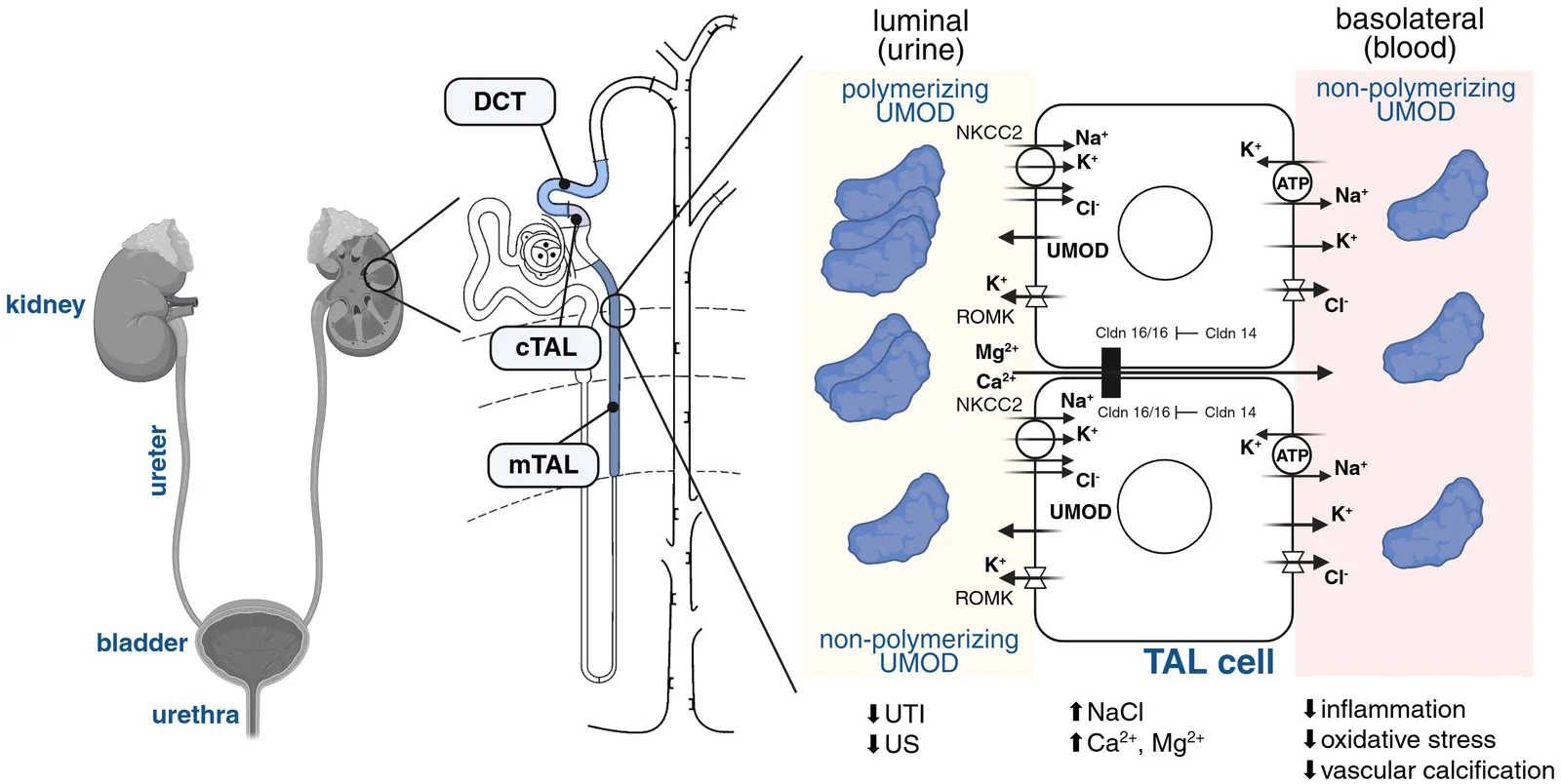
Synthesis and functions of uromodulin. Uromodulin (Umod) is produced mainly in the medullary (mTAL) and cortical thick ascending limb (cTAL) with minor contribution of the ensuing distal convoluted tubule (DCT). The apical Umod release recruits hepsin-mediated proteolytic cleavage producing a Umod variant capable of polymerization. The basolateral Umod secretion generates a non-polymerizing Umod variant. The urinary (polymerizing) Umod protects against the urinary tract infections (UTI) and calcium oxalate urinary stone (US) formation. The basolateral (non-polymerizing) Umod exerts anti-inflammatory and antioxidant effects in the renal interstitium and systemic circulation, thereby preventing vascular calcification. The intracellular Umod contributes to the transport function of the thick ascending limb (TAL).

Thus, the spectrum of physiological Umod functions is defined by the compartments where the protein acts, i.e. intracellular, interstitial, serum, or urine (**Figure 1**).

## Transcriptional regulation of uromodulin

The Umod expression is strongly modulated by single nucleotide polymorphisms (SNPs) located within a tightly inherited linkage disequilibrium block of the *UMOD* gene promoter (37–39). The *UMOD* promoter features two main genetic profiles in the modern human population, the ancestral and the derived haplotypes. The ancestral haplotype comprises the rs13333226-A, rs12917707-G, and rs4293393-A alleles representing the original evolutionary gene variant represented at the highest frequency in the modern population (40). The derived haplotype emerged later in human history and is less frequent. This haplotype comprises the mutated rs13333226-G, rs12917707-T, and rs4293393-G alleles (40). The ancestral haplotype is associated with significantly higher Umod expression compared to the derived one (37). While the ancestral haplotype provides better protection against UTIs, it has also been associated with higher risk of renal and cardiovascular diseases (37–42). Expression of the UMOD gene is governed by a number of transcription factors, among which the Hepatocyte Nuclear Factor 1-beta (HNF1B) plays the major role (43–46).

## Effects of aging on serum and urinary uromodulin levels

Aging is associated with progressive reduction of serum Umod levels largely reflecting the age-related tubular atrophy and gradual decline in the nephron number (47–49). Notably, despite generally lower serum Umod concentrations in older adults, individuals maintaining relatively higher Umod levels were at lower risk for rapid decline of the kidney function or developing the end-stage renal disease (ESRD) (49). The urinary Umod levels decrease during aging as well. Except few reports of unchanged or even increased urinary Umod excretion in aged individuals (50, 51), the vast majority of conducted studies showed lower urinary Umod levels in older compared to younger adults (12, 52–55). Notably, the only study reporting enhanced urinary Umod in aged individuals used proteomics for detection of urinary proteins and peptides (51), whereas various immunoassays were utilized in studies reporting reduced urinary Umod levels in older adults (12, 52–55). Thus, the discrepant results may be at least partially related to different methodology. Urinary uromodulin levels are inversely correlated with the UTI incidence in human. Analysis of older hospitalized patients enrolled in the Cardiovascular Health Study revealed a strong, albeit non-significant trend for a negative correlation between urinary Umod levels and the UTI risk (48, 56). A recent Mendelian randomization study enrolling over 40,000 of European and US participants showed that genetic Umod variants predicting higher urinary Umod levels were associated with reduced risk of upper UTIs (57). Along the same line, ancestral *UMOD* gene variants common in the modern population provide higher Umod expression and urinary excretion compared to less frequent derived *UMOD* gene variants, which is suggestive of positive evolutionary pressure towards better protection against UTIs (37). Accordingly, urinary Umod levels display inverse correlations with clinical UTI markers such as urinary leukocytes (37). Therefore, increased risk of UTIs in older individuals may be related to lower Umod production.

## Endocrine regulation of uromodulin in aging

Since Umod has been implicated in the transport functions of TAL and DCT, the sites of its production, hormones regulating these nephron segments are expected to affect the Umod expression and release as well.

### Hypothalamic-pituitary axis

The hypothalamic-pituitary hormone vasopressin (VP), also known as the antidiuretic hormone, is the major player in the endocrine regulation of TAL (58–61). TAL cells express the vasopressin V2 receptor (V2R) residing in their basolateral membrane. V2R signaling recruits the cyclic adenosine monophosphate (cAMP) as the second messenger with ensuing activation of the protein kinase A (PKA), which promotes the apical trafficking and phosphorylation of NKCC2 (59, 61). Experimental studies in transgenic mice, isolated TAL tubules, and cultured cells suggested that Umod facilitates NKCC2 activity thereby supporting the NaCl reabsorption along TAL (32, 33, 39). Further experimental work conducted in Xenopus oocytes and mice demonstrated activating effects of Umod on ROMK, which is a potassium channel mediating apical K^+^ recycling mandatory for the intact NKCC2 function in TAL (34). Notably, ROMK activity has been shown to stimulate the proteolytic processing and urinary release of Umod by TAL cells, which is suggestive of reciprocal activating interactions between the two proteins (35). The molecular mechanisms connecting Umod with NKCC2 or ROMK may be related to their interactions within the apical lipid rafts, since GPI-anchored proteins such as Umod appear to be generally involved in organization and stabilization of lipid rafts (62, 63). Experiments in rodents and cultured cells suggest that VP promotes assembly and apical trafficking of lipid rafts containing Umod and NKCC2 (32, 62). VP has further been shown to acutely stimulate the luminal Umod secretion in mice and cultured cells, as judged by effects of a selective V2R agonist desmopressin (dDAVP) (64). Similarly, water deprivation for 24 h led to enhanced urinary Umod excretion in mice (65). The resulting increase in urinary Umod levels may help to maintain improved protection against UTI during the VP-induced urinary concentration. Moreover, urinary Umod may interfere with endocytosis of the aquaporin 2 (AQP2) thereby promoting water reabsorption in the collecting duct synergistically with VP (65). In contrast to the short-term effects of water derivation or dDAVP treatment, chronic stimulation of V2R in VP-deficient Brattleboro rats using dDAVP infusion for several weeks was associated with a moderate decline in urinary Umod levels suggesting different adaptations of TAL to acute vs. chronic activation of the VP-V2R axis (66).

Human studies showed a trend towards a negative correlation between serum VP levels estimated by its surrogate marker copeptin and urinary Umod levels in healthy adults (67). The urine flow but not VP has been established as the dominant stimulus for the urinary Umod secretion in the human species (67). This mechanism may help to maintain optimal protection against UTI despite physiological fluctuations of the urine flow. Nevertheless, the urinary Umod excretion positively correlates with the urinary osmolality and this association is independent on VP (67). Thus, VP and Umod may synergistically promote the urinary concentration in humans, as has been suggested by experimental animal studies (32, 34, 67). Notably, aged individuals frequently exhibit elevated serum VP levels but decreased GFR (68). Thus, reduced urinary Umod excretion in older individuals may be primarily related to the age-dependent decline in GFR. Sustained elevations of circulating VP have been associated with hyperfiltration in diabetic patients and may provoke hyperfiltration of the remaining nephrons in older individuals as well (68, 69). Whether alterations in circulating VP levels substantially affect the Umod production and urinary excretion in older individuals remains to be clarified.

### Adrenal glands

The UMOD promoter region contains a predicted glucocorticoid response element (GRE) which is responsive to glucocorticoid exposure in luciferase reporter assays *in vitro* (39). The ancestral T Umod allele conserves the GRE and is associated with enhanced Umod expression in cultured cells along with elevated urinary Umod levels in humans, as compared to the derived C Umod allele with a single-nucleotide polymorphism disrupting GRE (39). Notably, the ancestral allele has been associated with higher risk for chronic kidney disease and hypertension, whereas the derived allele is protective against these risks (38, 39). Glucocorticoids have been shown to facilitate the transport activity of TAL but whether this effect depends on stimulation of Umod expression is unclear (70). In this context, Umod could contribute to the renal salt reabsorption via promoting NKCC2 activity, as suggested by animal and human studies (32, 39). Older adults generally display higher cortisol levels compared to the younger adults but the urinary Umod concentrations reported in aged individuals are mostly decreased (48, 71). Hence, effects of glucocorticoids on the Umod expression in the aged kidney require further characterization.

### Pancreas

Direct effects of the pancreatic hormones on the Umod expression and its serum or urinary levels have not been examined to date. However, an inverse correlation between serum Umod levels and insulin resistance has been demonstrated in cardiovascular patients by the Homeostatic Model Assessment of Insulin Resistance (HOMA) test (72). Along the same line, serum Umod showed negative correlations with fasting glucose and hemoglobin A1C (HbA1c) levels, as well as the diabetic status, particularly in men (72). In contrast to the serum Umod, urinary Umod levels have been shown to increase in diabetic patients, especially before development of microalbuminuria (73). Diabetes Mellitus significantly alters the renal function with frequent hyperfiltration in the early phase of diabetic kidney disease followed by reduction of the estimated glomerular filtration rate (eGFR) due to progressive nephron loss (74–76). Umod production and urinary levels demonstrate significant positive correlations with eGFR thus reflecting tubular functionality (12, 16, 42, 48). In this context, changes of the urinary Umod levels reported in diabetic patients may reflect hyperfiltration or deteriorations of the renal function rather than altered regulation of Umod production by insulin (42). However, experimental studies in isolated rodent TAL tubules demonstrated direct activating effects of insulin of the NaCl transport suggesting that Umod expression may be regulated by the hormone as well (77, 78). Moreover, hyperinsulinemia has been shown to increase fractional distal sodium reabsorption in hypertensive patients, while serum and urinary Umod display correlations with both hypertension and diabetes (39, 42, 48, 72, 79, 80). Thus, insulin has been shown to promote the TAL transport function but its direct effects on the Umod expression and release require further mechanistic studies.

Another major pancreatic hormone, glucagon, is an established activator of TAL as well (59, 81–83). However, direct effects of glucagon on Umod have not been studied to our knowledge. Diabetic patients, particularly those with the type 2 diabetes mellitus, exhibit paradoxically increased serum glucagon levels but whether the hormone directly affects serum or urinary Umod levels remains to be clarified (84–86). Notably, SNPs in the Umod promoter region appear to affect the risk of diabetic kidney disease suggesting that Umod may modulate the diabetic kidney damage (41). Differential effects of Umod gene variants on the glucose metabolism and blood pressure may be particularly relevant in older adults, since aging is associated with increasing incidence of diabetes and hypertension (39, 41, 87–89).

### Thyroid glands

Thyroid hormones, primarily triiodothyronine (T_3_) and thyroxine (T_4_), govern the energy metabolism of TAL thereby critically affecting its structure and transport capacity (90–93). Notably, experimental hypothyroidism in rats was associated with significantly reduced Umod expression and urinary excretion (91). The thyroid hormones may regulate the Umod expression via the epidermal growth factor (EGF) signaling (94). Experimental studies in rodents and cultured cells suggested that EGF promotes the nuclear translocation and activity of HNF1B, while the latter has been shown to facilitate the *UMOD* gene expression (43–46). EGF is produced and released into the urine by cells of TAL and DCT, which are also equipped with the EGF receptor (EGFR) at their apical membranes enabling the autocrine and paracrine EGF signaling modes (95, 96). In this context, decreased EGF signaling due to genetic deletion of cyclooxygenase 2 (COX-2) is associated with reduced Umod expression in mice (97). The urinary Umod and EGF levels display a moderate correlation and both have been increasingly recognized as biomarkers of the distal nephron functionality or tubular damage (98). Similar to Umod, the urinary excretion of EGF decreases with age (99). Furthermore, aging is associated with a gradual decline in thyroid axis activity reflected by a moderate decrease of T3 levels and peripheral response to the hormone alongside moderately enhanced thyroid-stimulating hormone (TSH) levels (100). Thus, suppression of the thyroid axis may contribute to decreased Umod production in aged individuals. However, effects of hypothyroidism or thyrotoxicosis on the urinary Umod levels in human have not been explicitly studied, to our knowledge.

### Parathyroid glands

Parathyroid hormone (PTH) has been implicated in the regulation of TAL transport function as well (101). Transcellular salt reabsorption via NKCC2 along with apical K^+^ recycling via ROMK creates a positive voltage at the luminal TAL side driving the paracellular reabsorption of divalent cations, primarily Ca^2+^ and Mg^2+^ (101, 102). PTH secretion is induced by decreases in serum Ca^2+^ levels to restore the systemic calcium homeostasis via release of Ca^2+^ ions from the bones and increased renal Ca^2+^ reabsorption. Experimental studies suggest that PTH stimulates NKCC2 activity via generation of intracellular cAMP and PKA activation (103). Further experimental studies in transgenic mice demonstrated that PTH facilitates the paracellular permeability of TAL for divalent cations as well, thereby substantially improving the renal calcium reabsorption (104). Although direct effects of PTH on the urinary Umod levels are not well documented, the hormone may indirectly modulate the Umod production via effects on the circulating Ca^2+^ concentrations. TAL cells express the calcium-sensing receptor (CaSR) residing in their basolateral membrane and adjusting the tubular Ca^2+^ reabsorption to the systemic calcium homeostasis (105). CaSR activation by increased interstitial Ca^2+^ levels reflecting the serum Ca^2+^ concentration inhibits NKCC2 and ROMK thereby reducing the transcellular drive for paracellular Ca^2+^ reabsorption. The latter is further suppressed by CaSR-dependent induction of the claudin-14 expression (106). Notably, studies in animals and healthy human subjects demonstrated a rapid reduction of the Umod secretion and urinary levels upon CaSR activation (107). Since Umod has been implicated in the regulation of TRPV5, reduced Umod release upon CaSR activation may serve to further limit the distal tubular calcium reabsorption (108). In addition to Ca^2+^, CaSR has been also shown to sense phosphate which inhibits the receptor activity (109). Older adults are notoriously susceptible to various deviations of calcium and phosphate homeostasis which may secondarily affect the Umod production, although these effects need to be characterized in more detail.

### Gonads

Sex hormones distinctly affect the Umod production. In general, urinary and serum Umod levels appear to be higher in women compared to men in spite of smaller kidneys and a lower total number of nephrons in women (12, 48, 110–112). This gender difference may be related to non-canonical or half estrogen response elements in the *Umod* gene (112, 113). While increased Umod levels in women may provide an additional protection against UPEC, thereby balancing the anatomical predisposition of women to UTI, decline in estrogen production during progress towards menopause may help to explain a more pronounced age-related decrease in urinary Umod levels in women compared to men (112, 113). Furthermore, experimental studies in rodents suggest that sex hormones affect the EGF signaling, although their effects on EGF and EGFR are complex and depend on the context (114–117). Therefore, decline in gonadal functions during aging may modulate the Umod expression directly or indirectly.

The available studies on endocrine regulation of the Umod expression, urinary Umod levels, as well as serum Umod concentrations are summarized in **Table 1**. However, these studies are not specifically focused on aging but rather present direct or (mostly) indirect evidence on endocrine Umod regulation in diverse physiological or pathophysiological settings. Therefore, effects of certain hormones on Umod in aged individuals may differ from their documented effects in younger adults or in patients with metabolic or endocrine disorders. As an example, a study by Trudu et al. is suggestive of stimulating effects of glucocorticoids on the Umod expression and urinary excretion (39). However, older adults frequently exhibit increased circulating cortisol levels but reduced urinary Umod excretion (48, 71). Thus, **Table 1** presents a summary of currently available data on the endocrine Umod regulation rather than specific effects of different hormones on Umod during healthy or pathophysiological aging. Future studies focused on the Umod regulation during aging are mandatory for improved understanding of the Umod biology.

**Table 1:** Putative effects of different hormones on the uromodulin expression, serum, and urinary levels, as reported by the available animal and human studies.

Hormone	mRNA	Serum	Urine	Species/model	(Patho)physiology	References
Vasopressin	↓	?	↑	M, R, C	Urine concentration	(64, 66)
Glucocorticoids	↑	?	↑	H, M, C	Hypertension, DM	(39)
Insulin	?	↓	↑	H	DM, pre-DM	(72, 73)
Triiodothyronine	↓	?	↓	R	Hypothyroidism	(91)
PTH	?	?	↑	M		(108)
Estrogens	↑	↑	?	H, M	Healthy women	(112)

C – cells, DM – diabetes mellitus, H – human, M – mouse, PTH – parathyroid hormone, R – rat, ↑ indicates a putative stimulation, ↓ indicates a putative suppression,? indicates lack of convincing information.

## Limitations

The growing interest for serum and urinary Umod as biomarkers of cardiovascular and renal diseases has been inspiring prospective and retrospective studies in various patient cohorts (48, 49, 72, 111, 118). However, only few of the available human studies specifically address the endocrine regulation of Umod (112, 113). Thus, the scarcity of human-derived data is the major limitation for the present synthetic review. Since aging is associated with gradual nephron loss and the respective decline in the functional TAL mass, it is difficult to dissect between effects of morphological renal changes vs. systemic endocrine alterations on the Umod production and release in aging. Similar limitations are applicable to the available experimental studies in animals or cell culture, i.e. only few of them specifically address aspects of the endocrine Umod regulation (64, 66, 91). Thus, effects of the aging-endocrine framework on the Umod production and release remain to be clarified in more detail.

## Conclusions

The endocrine regulation of Umod production and release in the aged kidney must be viewed in the context of tubular atrophy, progressive loss of nephrons, and altered tubular functionality including the responsivity to endocrine stimuli. While serum levels of VP and glucocorticoids typically rise with aging, the renal response to these hormones may be blunted instead, as suggested by experimental studies in rodents (68, 119–121). Along the same line, reduced nephron number limits the renal Umod production (122). The age-dependent alterations in the endocrine system may aggravate the reduction in serum or urinary Umod levels. For example, decreased circulating estrogen levels in post-menopausal women may help to explain a more pronounced decrease of urinary Umod levels in aged women compared to men (112, 113). Since endocrine and metabolic disorders such as diabetes mellitus or hypothyroidism disproportionally affect older adults, the disease-related hormonal imbalance may additionally influence the Umod production during pathophysiological aging (100, 123, 124). It would be interesting to evaluate effects of lifestyle or pharmacological corrections of hormonal imbalance on serum and urinary Umod levels in older adults. In view of numerous clinically relevant aspects associated with Umod biology, improved understanding of its regulation may be instrumental for strategies of healthy aging.

## Review methodology

The PubMed database was searched for original and review articles published through 22.07.2026 using combinations of the following keywords and MeSH terms: “uromodulin”, “Tamm–Horsfall protein”, “Tamm Horsfall”, “THP”, combined with hormone names to capture interactions with diverse endocrine axes. Search threads used Boolean operators and truncations where appropriate (for example: uromodulin OR “Tamm Horsfall” OR THP) AND (vasopressin OR ADH OR “antidiuretic hormone”). The search was limited to PubMed-indexed articles and did not expand to other bibliographic databases.
